# Variability of Leser-Trélat Sign Secondary to Melanoma In Situ

**DOI:** 10.7759/cureus.53639

**Published:** 2024-02-05

**Authors:** Olivia C Silveri, Franklin James, Brian Dickens

**Affiliations:** 1 Dermatology, Edward Via College of Osteopathic Medicine, Blacksburg, USA; 2 Family Medicine, Carilion Clinic, Roanoke, USA

**Keywords:** cutaneous malignancy, seborrheic keratoses, variability, leser-trélat sign, melanoma

## Abstract

Leser-Trélat sign (LTS) is characterized as an eruptive display of multiple seborrheic keratoses (SKs) in association with malignancy. This case highlights the variable presentation of LTS secondary to melanoma. To our knowledge, this LTS pattern is the first case where the sign manifests as a subtle pattern secondary to melanoma. This stands in contrast to the five documented cases in the literature of LTS-melanoma, which exhibited distinctive and eruptive patterns.

A 64-year-old Caucasian female presented for a wellness examination. No personal history of skin cancer was noted. Patient displayed an onset proliferation of SKs with an irregular, sub-centimeter macular nevus over her right lateral mid back. A 6mm punch biopsy was significant for melanoma in situ, arising within a lentiginous compound dysplastic nevus, focally abutting one peripheral tissue edge. A re-excision with a minimum of 5mm margins was completed and the specimen was negative for residual in situ melanoma.

Because of the rare occurrence of this delicate pattern at the site of the melanoma, this presentation adds to the knowledge surrounding this diagnosis. This case emphasizes the importance of maintaining vigilance regarding skin manifestations associated with disease and highlights the critical importance of observation and identification of subtle physical exam findings.

## Introduction

There have been few cases of Leser-Trélat sign (LTS) in the literature, specifically associated with melanoma [1-5]. LTS is defined as a paraneoplastic syndrome with acute onset of multiple seborrheic keratoses (SKs), a cutaneous marker that is secondary to primary tumor or malignancy [1,5-8]. LTS was first characterized in the 1800s by Edmund Leser and Ulysse Trélat, as a cutaneous pattern with sudden eruption of cherry angiomas in oncologic patients, with additional descriptions by Hollander in 1900 regarding the appearance of seborrheic keratoses [1,9,10]. Pathogenesis of the Leser-Trélat sign is hypothesized to stem from release of epidermal growth factor (EGF) and transforming growth factor alpha (TGF-a) from additional malignant cells and keratinocyte proliferation [1,4,11]. Another hypothesis includes unidentified growth factors from malignancy, such as polypeptide growth factors capable of binding EGF receptors without cross-reactivity to anti-EGF antibodies [9].

LTS is most commonly linked with gastrointestinal tract tumors (72%) and adenocarcinomas (76%). Over 60% of cases associated with LTS are described in advanced stages of primary malignancy or metastases [4]. Rare cases may present with breast, liver, pancreas, bladder, kidney ovary, prostate, nasopharyngeal carcinoma, uterus, lymphoma, melanoma, and lung cancer [1,6,11,12]. To our knowledge, there have only been five cases documented on LTS secondary to cutaneous melanoma [1-5].

## Case presentation

A 64-year-old female presented to the primary care office for a routine wellness examination. She had no complaints at the time of her visit. Past medical history was significant only for migraine headaches and exercise-induced asthma. No personal history of skin cancer was noted. Screenings for breast, cervical, and colon cancer were either up to date or a plan for screening was made at the time of her wellness visit. Family history was significant for lung cancer (mother) and pharyngeal cancer (brother), and squamous and basal cell carcinomas (father). The patient had an 11-pack-year smoking history, having quit at the age of 27. She grew up in Texas and California earlier in life and had extensive sun exposure. 

On physical examination, she was noted to have a proliferation of seborrheic keratoses on her back, which was a change compared to her previous wellness visit. Aware of the reported correlations of LTS with an underlying cutaneous melanoma (Table 1), and having seen this correlation previously, her physician screened her skin for irregular nevi. 

**Table 1:** The five known cases of Leser-Trélat sign (LTS) secondary to melanoma and the distinct presentations of the lesions.

Author	Patient age, sex	Leser-Trélat sign (presentation array, location)	Primary malignancy (Breslow thickness)
Gori N et al. (2020) [1]	80-year-old woman	Trunk described as “Christmas tree pattern”, head and neck	Melanoma on right flank, 4.5mm
Ellis DL et al. (1987) [2]	54-year-old man	Upper chest and back	Melanoma on lower back, 0.75mm
Fanti PA et al. (1989) [3]	69-year-old man	Trunk, thighs, described as “Christmas tree pattern”	Nodular melanoma on left thigh, 2.16mm
Siedek V et al. (2009) [4]	82-year-old man	Head and trunk, described as “Christmas tree pattern”	Unknown with metastatic involvement of parotid gland and cervical lymph node
Pereira R et al. (2019) [5]	71-year-old woman	Right posterior thoracic region	Unknown with metastatic involvement of pleura and axilla lymph nodes

**Figure 1:**
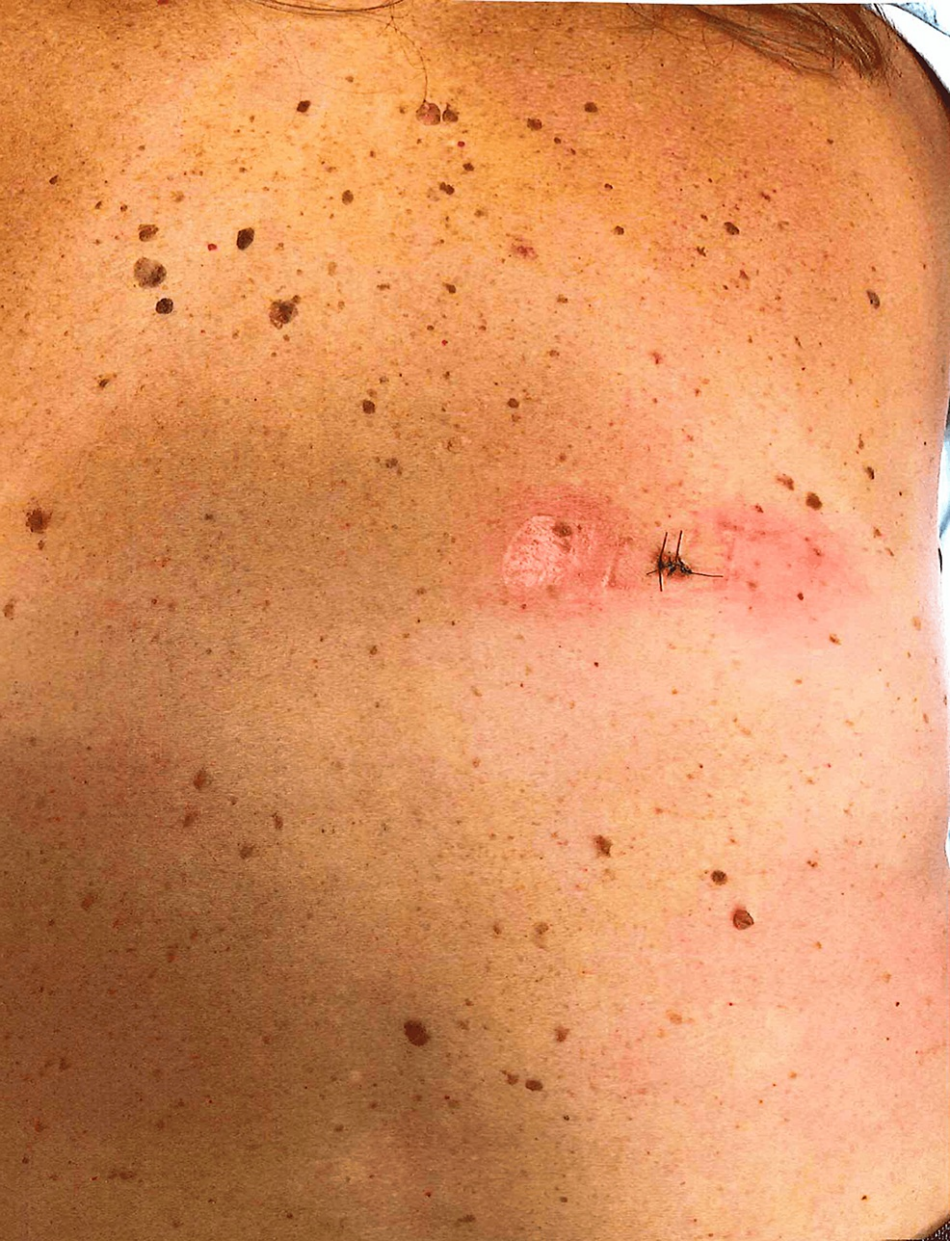
6mm punch biopsy, positive for in situ malignant melanoma

**Figure 2:**
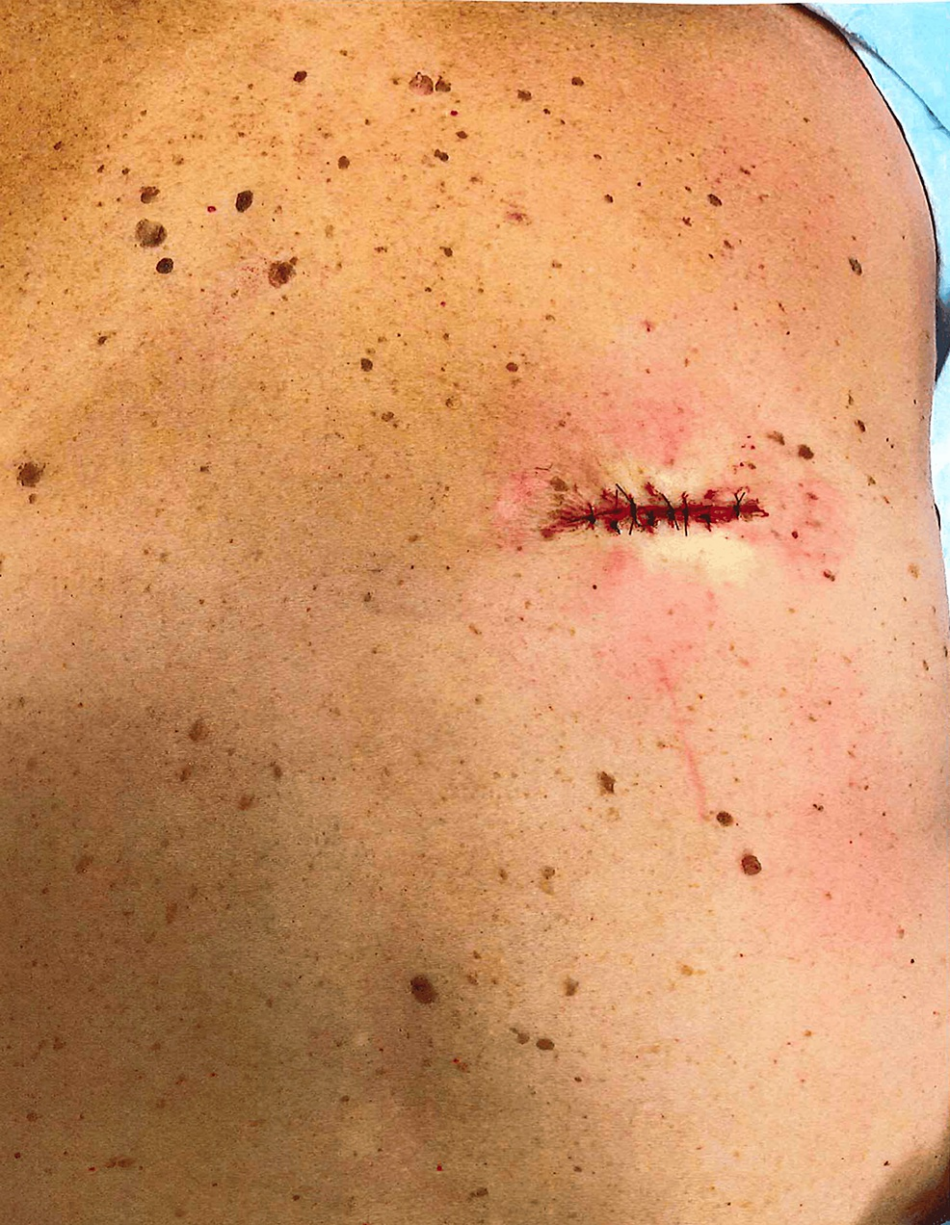
Re-excision with minimum of 5mm margins was completed one week later Specimen was found to be negative for residual in situ malignant melanoma

This case is unlike these previously published LTS-melanoma reports and presents similarly to the discrete pattern shown in adenocarcinomas [13,14]. An irregular, sub-centimeter macular nevus was noted over patient’s right lateral mid back with irregularity of color and border noted under dermatoscopic examination and the decision was made to have the patient return for a 6mm punch biopsy (Figure 1). The subsequent biopsy showed histological characteristics of comet-like shape melanoma in situ, arising within a lentiginous compound dysplastic nevus, focally abutting one peripheral tissue edge. A re-excision with a minimum of 5mm margins was completed one week later and the specimen was found to be negative for residual in situ malignant melanoma (Figure 2). 

The patient was subsequently referred to dermatology to establish additional regular monitoring. The importance of further sun protection was also emphasized. 

## Discussion

This case supports the importance of the identification of subtle cutaneous exam findings, especially with LTS, a cutaneous indicator of underlying malignancy.

In the previously documented cases of melanoma with associated LTS, the LTS pattern was a definite spread of prominent maculopapular nodular SKs [1-5]; while this case is a pattern of subtle macular SKs similar to other malignancies such as adenocarcinoma [13,14]. This exemplifies the variability of the LTS and highlights a distinct subtle pattern with melanoma. Though only a few instances of this correlation have been presented in the medical literature, a skin screening examination is an easy measure to implement in the setting of potential LTS.

It is recommended for patients with any eruption of lesions to undergo skin screenings, as early clinical diagnosis will aid in prompt treatment of a primary malignancy and support individual vigilance and health maintenance. It has been noted in the literature that screening with dermatoscopy improves sensitivity in detection of seborrheic keratosis-like melanoma [1,15], and therefore a dermatoscope was used in our case. Spontaneous regression of seborrheic keratoses may occur following tumor excision or reduction post-treatment, which may be a factor in diagnosing LTS [4]. Patients presenting with the pattern of LTS are considered to be harboring malignancy until additional testing and confirmation prove otherwise [6]. However, chronic presentations with similarities to LTS patterns without malignancies have raised questions regarding the existence of LTS and continue to be explored in literature [12]. 

Physicians with insight into the variability of LTS, rather than the previously reported patterns, will be more vigilant of findings that will lead to a higher index of suspicion for possible associated malignancies. 

## Conclusions

Despite the rarity of the concurrent presentation of melanoma and LTS, we recognize that physicians with insight into the variability of LTS may have a high index of suspicion and a higher chance of diagnosing this pattern. This is crucial as physicians can utilize non-invasive screening tools, such as dermatoscopy, for prompt diagnosis and treatment of potential cancerous lesions. Awareness is the key to aiding in the knowledge of early clinical diagnosis and advancing individual health maintenance and yearly skin cancer screenings.
